# Global taxonomic and functional patterns in invertebrate assemblages from rocky-intertidal mussel beds

**DOI:** 10.1038/s41598-023-50549-8

**Published:** 2024-01-02

**Authors:** Nicole M. Cameron, Ricardo A. Scrosati, Nelson Valdivia, Zechariah D. Meunier

**Affiliations:** 1 Department of Biology, St. Francis Xavier University, Antigonish, NS B2G 2W5 Canada; 2 Instituto de Ciencias Marinas y Limnológicas and Centro FONDAP de Investigación de Dinámicas de Ecosistemas Marinos de Altas Latitudes (IDEAL), Universidad Austral de Chile, 5090000 Valdivia, Chile; 3 Department of Integrative Biology, Oregon State University, Corvallis, OR 97331 USA

**Keywords:** Ecology, Zoology

## Abstract

Mussels form extensive beds in rocky intertidal habitats on temperate seashores worldwide. They are foundation species because their beds host many invertebrates. Mussels and their associated species differ taxonomically among biogeographic regions, but all mussel beds exhibit similar structural and functional properties. Therefore, we investigated if rocky-intertidal mussel beds from around the globe host associated communities that are functionally similar despite their underlying taxonomic differences. We gathered datasets on the abundance of invertebrates found in rocky-intertidal mussel beds from the eastern and western boundaries of the Pacific and Atlantic Oceans from both hemispheres and, then, we compared their taxonomic and functional properties. Taxonomic composition differed markedly among coasts when analyzed at the taxonomic resolution reported by the surveys (often species). However, taxonomic groups with similar ecologies (28 groups including barnacles, decapods, gastropods, polychaetes, etc.) were more universally present in mussel beds. Concomitantly, functional categories of trophic level, body type, and mobility were almost always present on all studied coasts. These taxonomic groups and trait categories, however, showed regional patterns based on their relative abundances. Overall, the ability of mussel beds to host a core community type based on taxonomic groups and functional traits emphasizes their importance for biodiversity and community functioning, making them critical organisms to preserve.

## Introduction

Foundation species are spatially dominant species whose body structures create complex habitats that provide shelter from abiotic and biotic stresses. They are critically important because of the diversity of species that their stands host thanks to those properties. Foundation species can be primary producers such as trees and seaweeds or animals such as bivalves and corals^1–4^. Recognition of their ecological relevance especially given recent losses due to anthropogenic factors^5–8^ is fueling the need to understand what kinds of associated communities are hosted by foundation species. This article presents the first global analysis of the taxonomic and functional diversity of invertebrate assemblages living in rocky-intertidal mussel beds.

**Figure 1:**
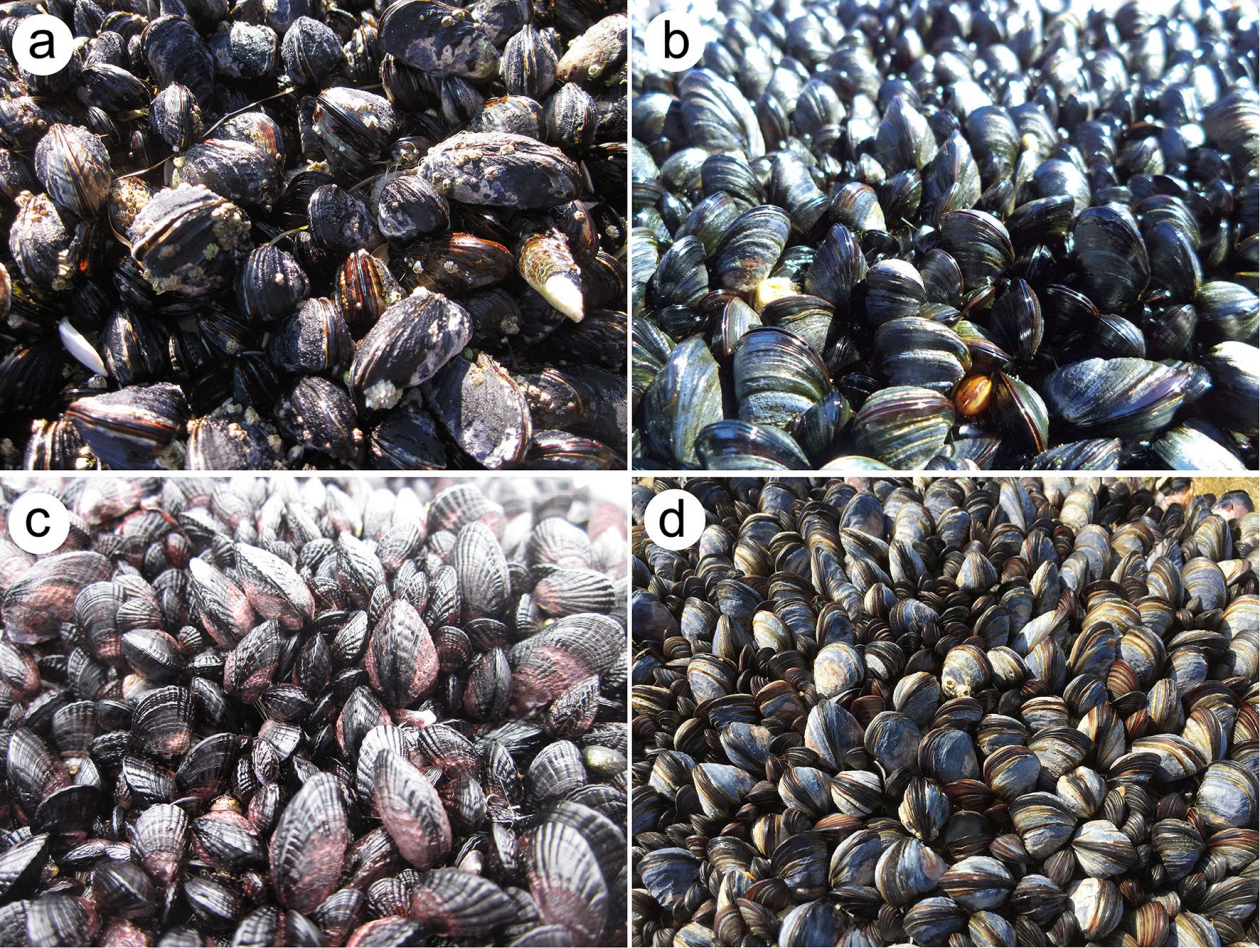
Rocky-intertidal mussel beds from (**a**) Oregon, United States (*Mytilus californianus* bed at Strawberry Hill), (**b**) Nova Scotia, Canada (*Mytilus edulis* and *M. trossulus* bed at Western Head), (**c**) Chile (*Perumytilus purpuratus* bed at Pucatrihue), and (**d**) Portugal (*Mytilus galloprovincialis* bed at Ericeira). Photos taken a low tide by RAS.

Mussels are bivalves that act as foundation species on temperate seashores across the world. In rocky intertidal habitats, they often occur at high densities covering extensive areas of the substrate, to which they remain attached with byssal threads^9–11^ (Fig. 1). The spaces among the mussels experience lower desiccation and thermal stress during low tides and lower hydrodynamic stress during high tides than open intertidal areas. At the same time, the matrix of mussel shells constitutes colonizable substrate that is more complex than the natural substrate. In addition, due to the limited water motion among the mussels, these stands passively accumulate mussel biowaste and external detritus and sediments, which small species use as food, safe habitat, or both^12–16^. Overall, these properties allow mussel beds to host many small species. For example, tens of species from several phyla have been identified in rocky-intertidal mussel beds on the NW^17^, SW^14^, NE^18^, and SE^19,20^ Pacific coasts and on the NW^21^, SW^22,23^, NE^24,25^, and SE^26^ Atlantic coasts. Naturally, those associated communities differ greatly in species composition, as distant biogeographic regions have different evolutionary histories and thus dissimilar species pools^27^. In other words, globally common patterns in such communities are not evident when viewed at the species level. However, given the common properties of rocky-intertidal mussel beds, global similarities in their associated communities might emerge if evaluated at a coarser taxonomic resolution, as species within broad groups are often ecologically similar. Ultimately, functional aspects of the associated communities may be responsive to the common properties of mussel beds and, thus, might also exhibit globally common patterns. Therefore, the present study investigates if rocky-intertidal mussel beds from temperate coasts around the globe host associated communities that share broad taxonomic and functional similarities despite taxonomic differences at the species level.

To address our objective, we focused on invertebrate assemblages, which are by far the main constituents of the communities living in mussel beds and the organisms that are typically reported by surveys. Specifically, we gathered datasets on the abundance of invertebrate taxa found in rocky-intertidal mussel beds from the eastern and western boundaries of the Pacific and Atlantic Oceans from both hemispheres. Then, we compared those assemblages based on their taxonomic and functional properties. Functional aspects of communities are often inferred based on functional traits of their constituent species. This is a common approach because it is generally impractical to measure various functional processes directly on all species in a community^28–31^. Therefore, to address our goal, we used information on functional traits for the invertebrate taxa that were listed in the abundance datasets that were available for this study (see “Methods”).

## Results

### Global taxonomic patterns

**Figure 2:**
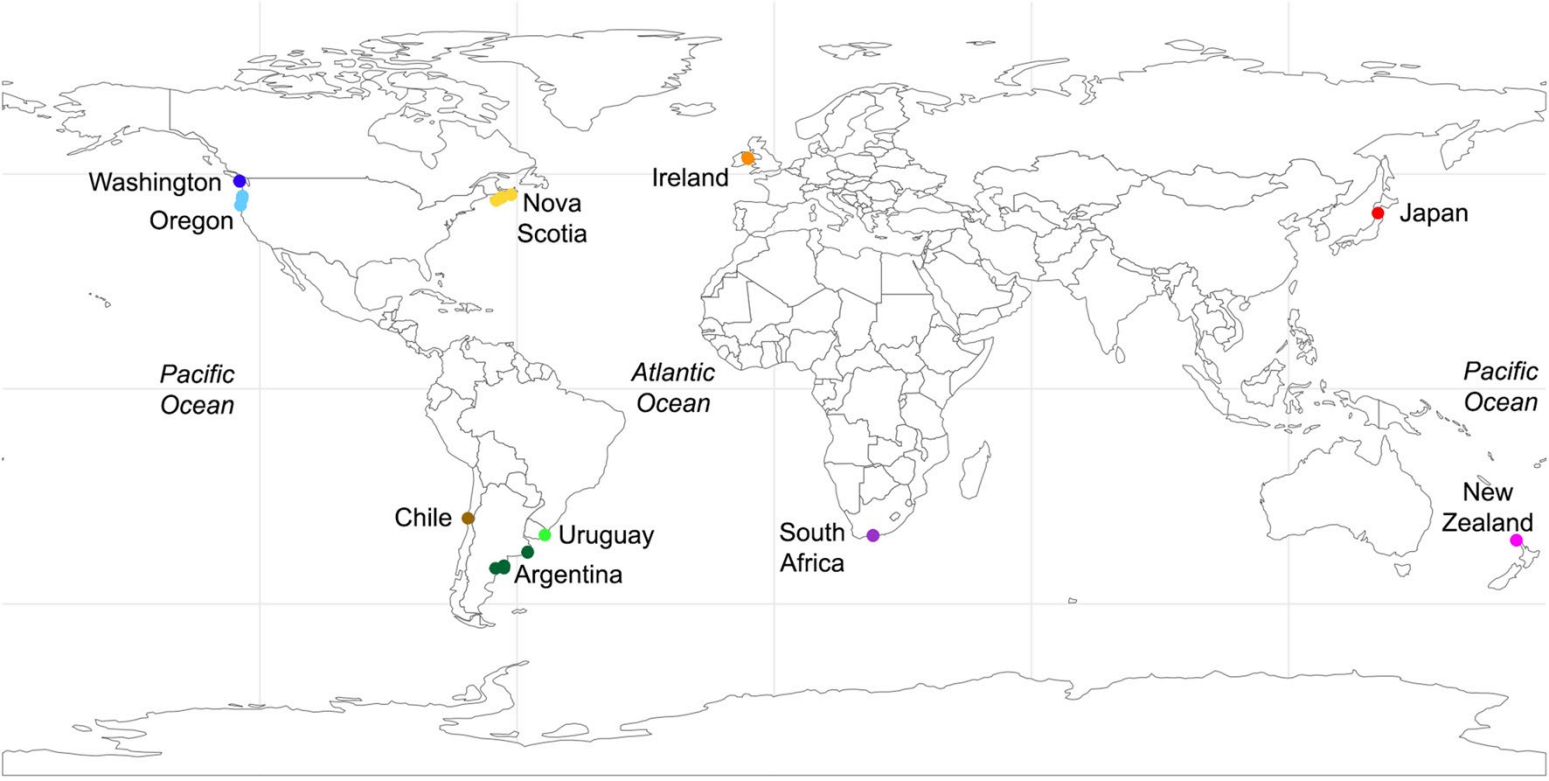
Map showing the coasts for which invertebrate abundance datasets were available for this study. The surveyed locations cover the NW, SW, NE, and SE coasts of the Pacific and Atlantic Oceans, which are temperate shores where rocky-intertidal mussels beds occur. The map was done by N. M. Cameron using R version 4.0.2 (www.R-project.org).

**Figure 3:**
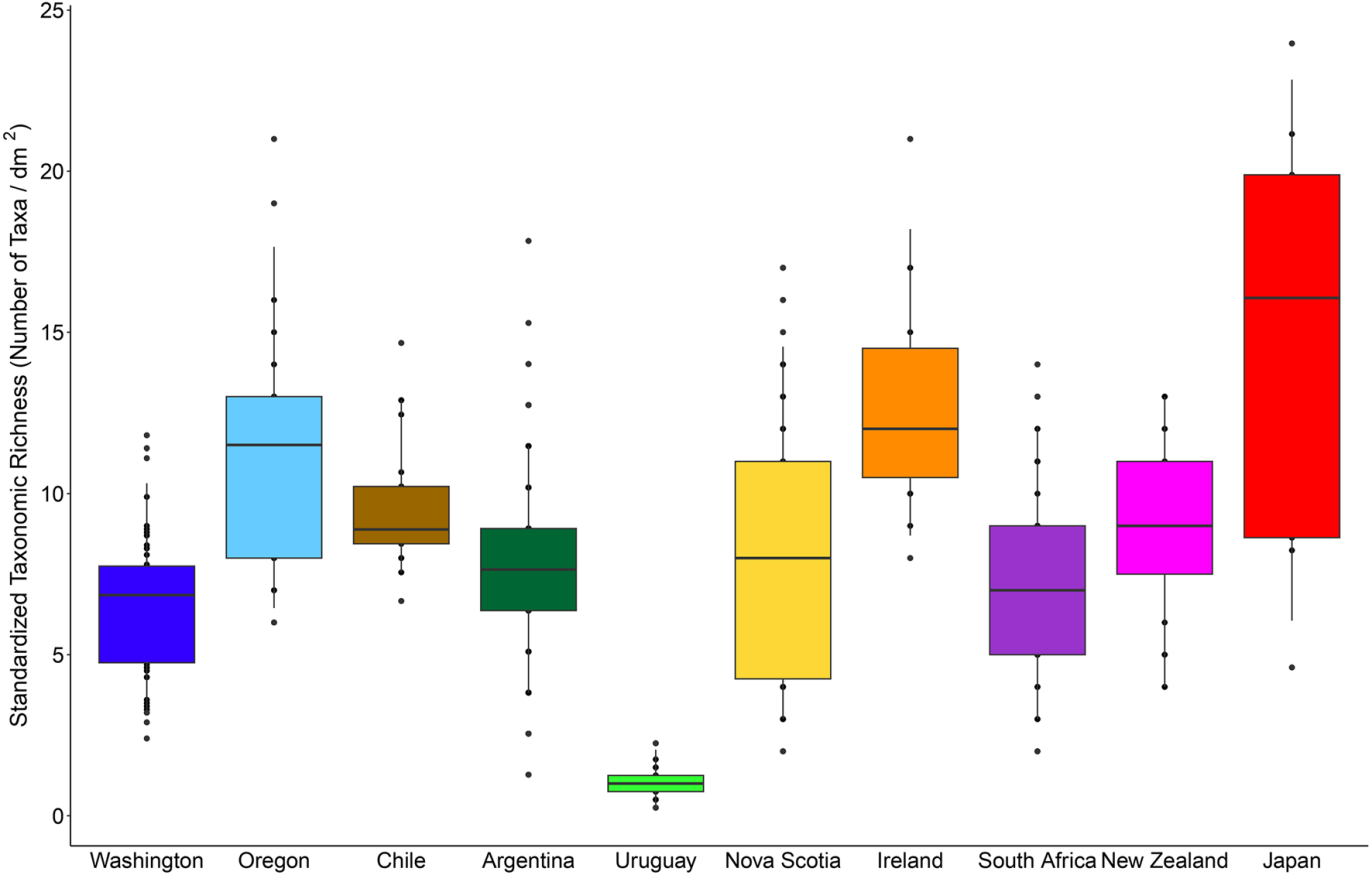
Standardized taxonomic richness (number of identified taxa per dm^2^). For each coast, the box contains the median and is delimited by the 25th and 75th percentiles of the data, while the whiskers are bound by the 5th and 95th percentiles.

The surveys that were available for this study (Fig. 2, Table 1) identified 413,446 organisms of 601 invertebrate taxa found in intertidal mussel beds from temperate rocky shores around the world. The highest number of identified taxa per survey (242) was reported for Washington and the lowest (26) for New Zealand (Table 1). The level of taxonomic resolution varied among the surveys, as the percentage of the reported taxa identified as species ranged from 13.8% in Uruguay to 84.7% in Washington (Table 1). Standardized taxonomic richness (number of identified taxa per unit area) varied statistically among the surveyed coasts (*F*_9, 606_ = 34.36, *P* < 0.001; Fig. 3), although values were relatively comparable among the coasts (Fig. 3). Only 7% of the taxa identified as species were reported for more than one coast.

**Table 1:** Sources and basic sampling properties of the datasets on invertebrate abundance from rocky-intertidal mussel beds that were available for this study.

Coast	Ocean boundary	Plot size (cm^2^)	Sample size (n)	Number of identified taxa	% of taxa identified as species	Number of identified species
Washington, USA	NE Pacific	1000	54	242	84.7	205
Oregon, USA	NE Pacific	100	30	40	65	26
Chile	SE Pacific	225	21	73	45.2	33
Argentina	SW Atlantic	78.5	288	55	50.9	28
Uruguay	SW Atlantic	400	29	29	13.8	4
Nova Scotia, Canada	NW Atlantic	100	90	50	64	32
Ireland	NE Atlantic	100	15	37	64.9	24
South Africa	SE Atlantic	100	60	67	71.6	48
New Zealand	SW Pacific	100	20	26	19.2	5
Japan	NW Pacific	35.2–521.3	9	30	83.3	25

See Supplementary Note for more details on each surveyed coast.

**Figure 4:**
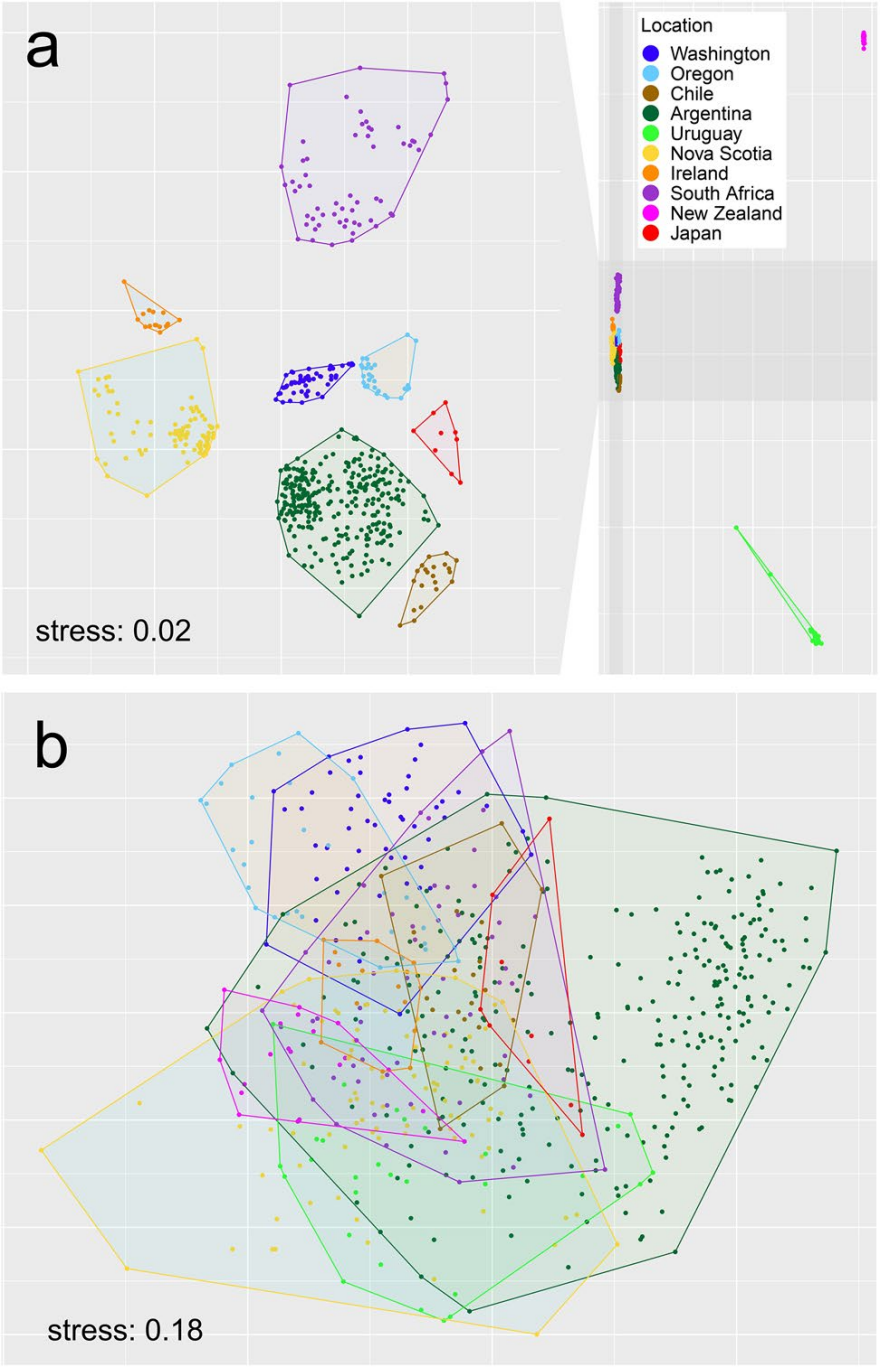
Nonmetric multidimensional scaling (NMDS) of all plots based on (**a**) the density of the taxa identified by the surveys and (**b**) the density of the 28 broad taxonomic groups listed in Table 2.

**Figure 5:**
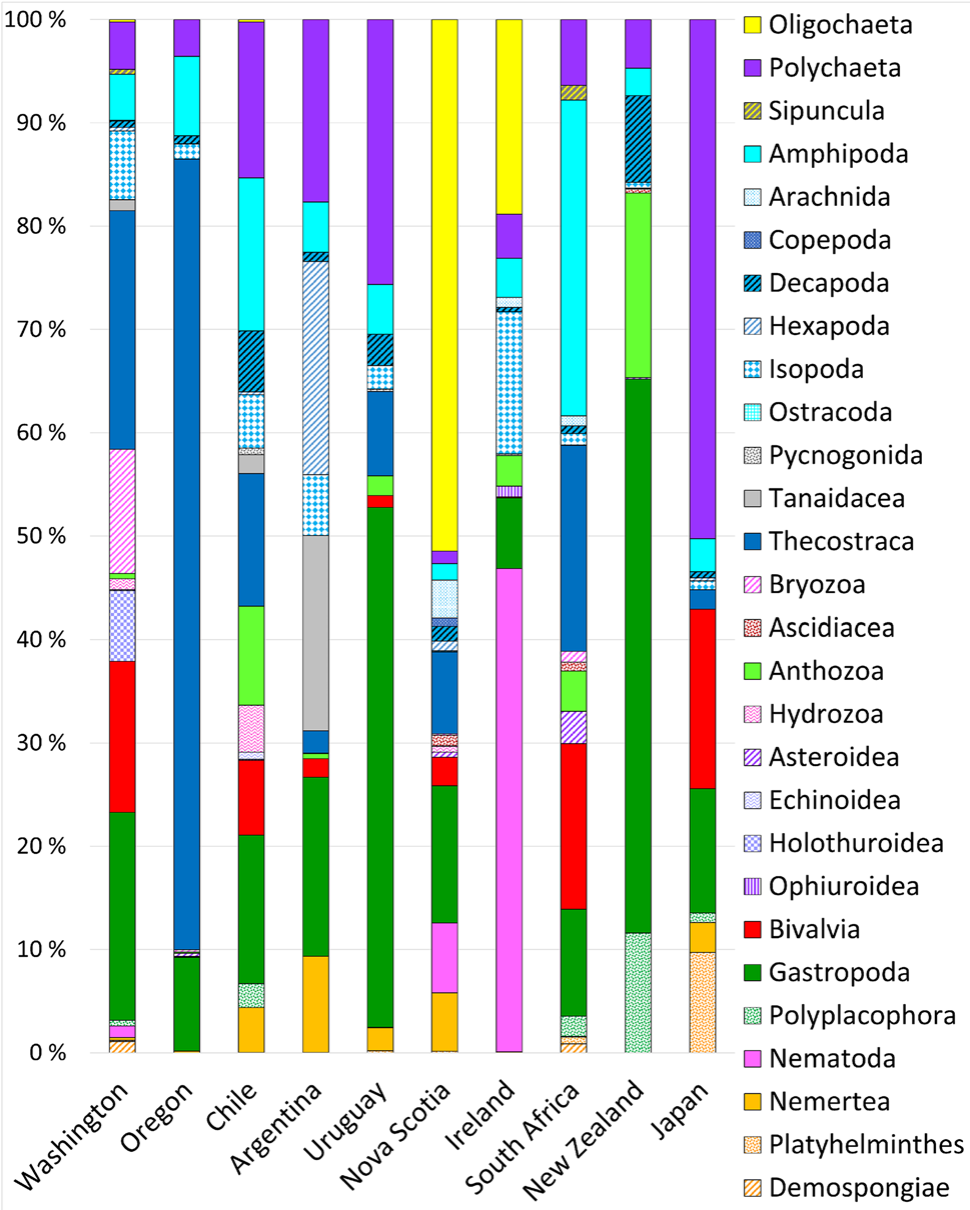
Mean relative (%) abundance of the 28 taxonomic groups listed in Table 2 for each surveyed coast.

Using the density values of the identified taxa (which included many species), the taxonomic composition of associated communities varied significantly among coasts (pseudo *F*_9, 606_ = 30.72, *P* < 0.001), with marked differences between coasts indicated by the NMDS ordination (Fig. 4). Using the density values of the 28 broad taxonomic groups listed in Supplementary Table S1 (see “Methods” for rationale), taxonomic composition also varied among coasts (pseudo *F*_9, 606_ = 28.88, *P* < 0.001), but the NMDS ordination showed overlaps among most coasts (Fig. 4). To evaluate how taxonomic groups characterize the associated communities globally, we calculated the relative density of each taxonomic group for each plot. Nine of these taxonomic groups (amphipods, anthozoans, barnacles, bivalves, decapods, gastropods, isopods, nemerteans, and polychaetes) were present on at least eight of the 10 surveyed coasts, while the other 19 taxonomic groups were present on seven or fewer coasts (Table 2). For most taxonomic groups, relative density differed among the surveyed coasts (Fig. 5, Supplementary Table S1). A few geographic patterns of interest emerged. For example, barnacles (Thecostraca) were most prominent on the three coasts surveyed in the eastern Pacific (Washington, Oregon, and Chile) as well as South Africa. Oligochaetes and nematodes, on the other hand, were relatively most abundant on the two coasts surveyed in the northern Atlantic (Nova Scotia and Ireland) (Fig. 5).

**Table 2:** Presence (X) and absence (–) of the 28 broad taxonomic groups reported for the ten surveyed coasts: Washington (WA), Oregon (OR), Chile (CH), Argentina (AR), Uruguay (UR), Nova Scotia (NS), Ireland (IR), South Africa (SA), New Zealand (NZ), and Japan (JA).

Group	Phylum	Common name of organisms	WA	OR	CH	AR	UR	NS	IR	SA	NZ	JA
Oligochaeta	Annelida	Oligochaetes	X	–	X	–	–	X	X	–	–	–
Polychaeta	Annelida	Polychaetes	X	X	X	X	X	X	X	X	X	X
Sipuncula	Annelida	Peanut worms	X	X	–	X	–	–	–	X	–	–
Amphipoda	Arthropoda	Amphipods	X	X	X	X	X	X	X	X	X	X
Arachnida	Arthropoda	Mostly mites, with a spider species reported in Japan and a pseudoscorpion species reported in South Africa	X	–	X	–	–	X	X	X	–	–
Copepoda	Arthropoda	Copepods	–	–	–	–	–	X	–	–	–	–
Decapoda	Arthropoda	Crabs	X	X	X	X	X	X	X	X	X	X
Hexapoda	Arthropoda	Insects	X	X	X	X	–	X	–	–	–	X
Isopoda	Arthropoda	Isopods	X	X	X	X	X	X	X	X	X	X
Ostracoda	Arthropoda	Ostracods	–	–	–	X	–	–	–	–	–	–
Pycnogonida	Arthropoda	Sea spiders	X	–	X	X	X	–	X	X	–	–
Tanaidacea	Arthropoda	Tanaids	X	–	X	X	–	–	–	–	X	–
Thecostraca	Arthropoda	Barnacles	X	X	X	X	X	X	–	X	–	X
Bryozoa	Bryozoa	Bryozoans	X	X	–	–	–	X	–	X	–	–
Ascidiacea	Chordata	Tunicates	X	–	–	X	–	X	–	X	X	–
Anthozoa	Cnidaria	Anemones and corals	X	X	X	X	X	X	X	X	X	–
Hydrozoa	Cnidaria	Hydrozoans	X	–	X	–	–	X	–	–	–	–
Asteroidea	Echinodermata	Sea stars	X	X	–	–	–	X	–	X	X	–
Echinoidea	Echinodermata	Sea urchins	X	–	X	–	–	–	–	–	–	–
Holothuroidea	Echinodermata	Sea cucumbers	X	X	–	–	–	–	–	–	–	–
Ophiuroidea	Echinodermata	Brittle stars	X	X	X	–	–	–	X	–	–	–
Bivalvia	Mollusca	Bivalves	X	X	X	X	X	X	X	X	–	X
Gastropoda	Mollusca	Snails	X	X	X	X	X	X	X	X	X	X
Polyplacophora	Mollusca	Chitons	X	X	X	–	X	–	–	X	X	X
Nematoda	Nematoda	Nematodes	X	–	–	–	–	X	X	–	–	–
Nemertea	Nemertea	Nemerteans	X	X	X	X	X	X	X	X	–	X
Platyhelminthes	Platyhelminthes	Flatworms	X	–	–	X	X	X	–	X	–	X

### Global functional patterns

**Figure 6:**
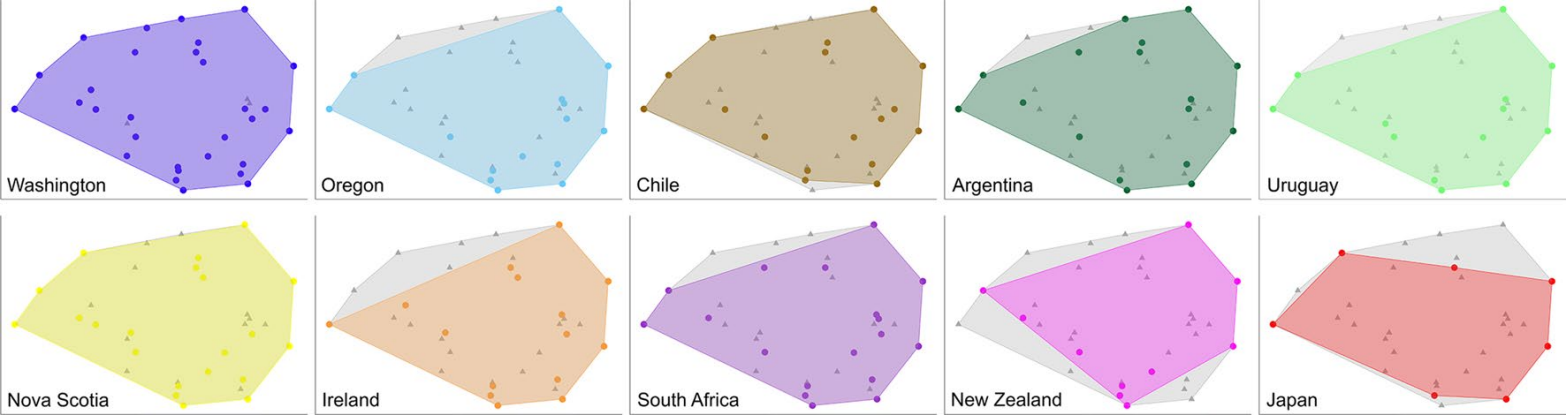
Polygons indicating the functional trait space for each surveyed coast based on the taxa identified at each coast (colored polygons) overlaid onto a grey polygon that indicates the functional trait space based on the taxa identified by all surveys.

**Figure 7:**
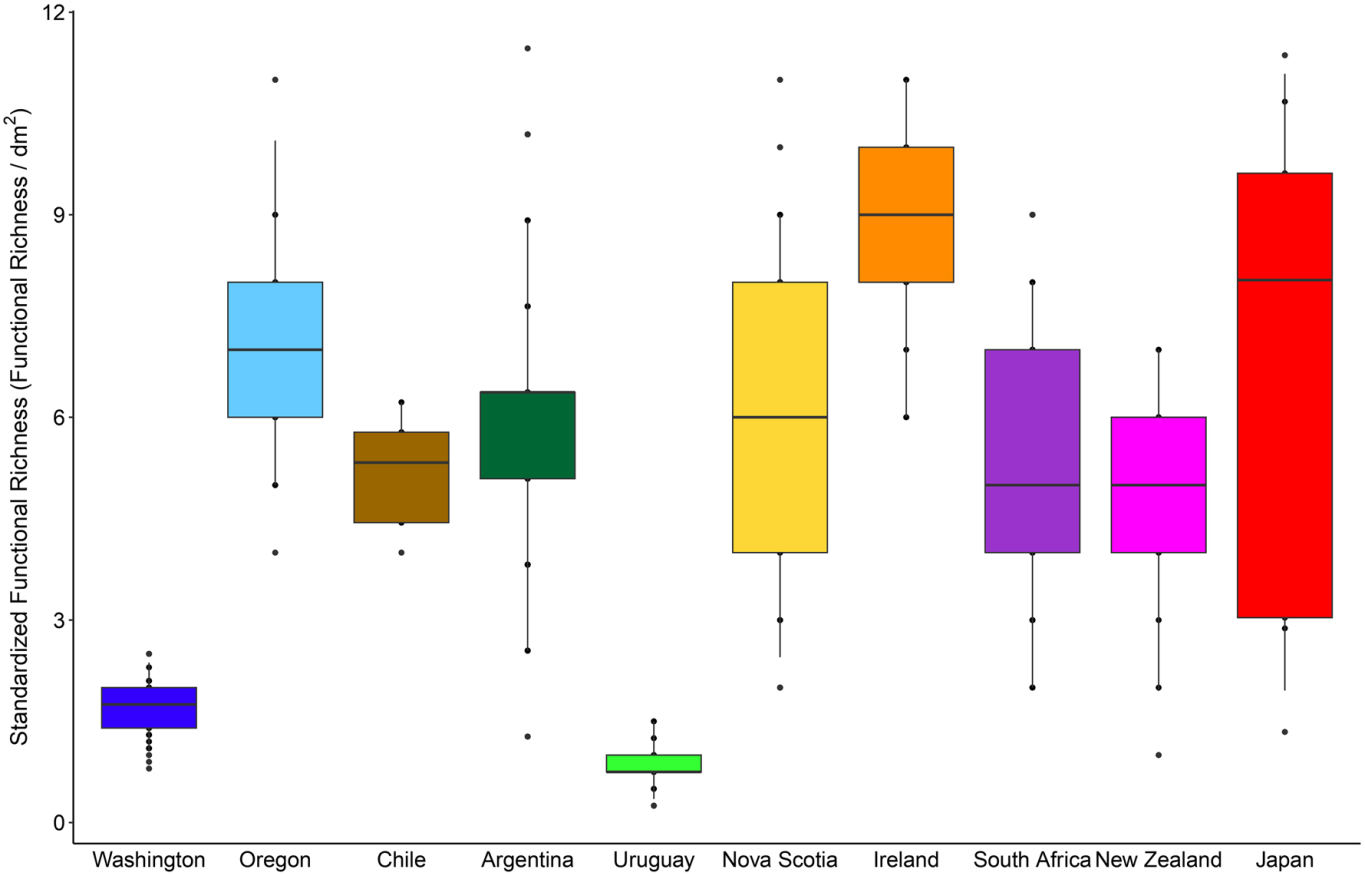
Standardized functional richness (functional richness expressed per dm^2^). For each coast, the box contains the median and is delimited by the 25th and 75th percentiles of the data, while the whiskers are bound by the 5th and 95th percentiles.

The trait space calculated using the trait categories determined for the taxa reported by the surveys was similar among the surveyed coasts (Fig. 6). Standardized functional richness (functional richness expressed per unit area) varied among the coasts (*F*_9, 606_ = 58.57, *P* < 0.001), although most coasts exhibited similar values (Fig. 7).

**Figure 8:**
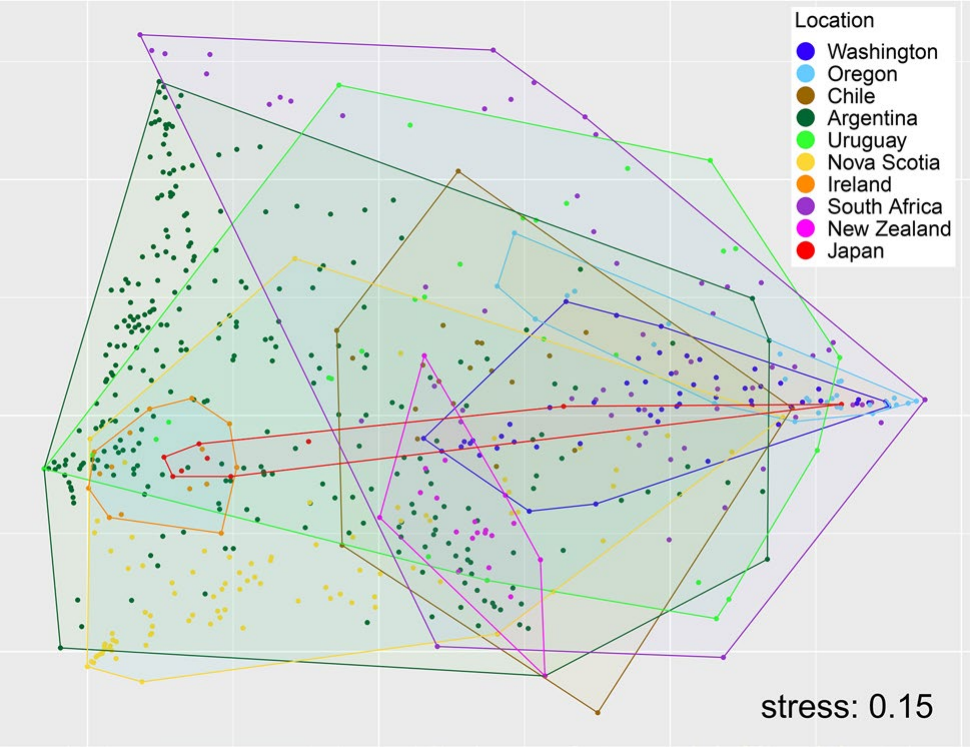
Nonmetric multidimensional scaling (NMDS) of all plots based on the relative abundance of all of the trait categories considered in this study.

**Figure 9:**
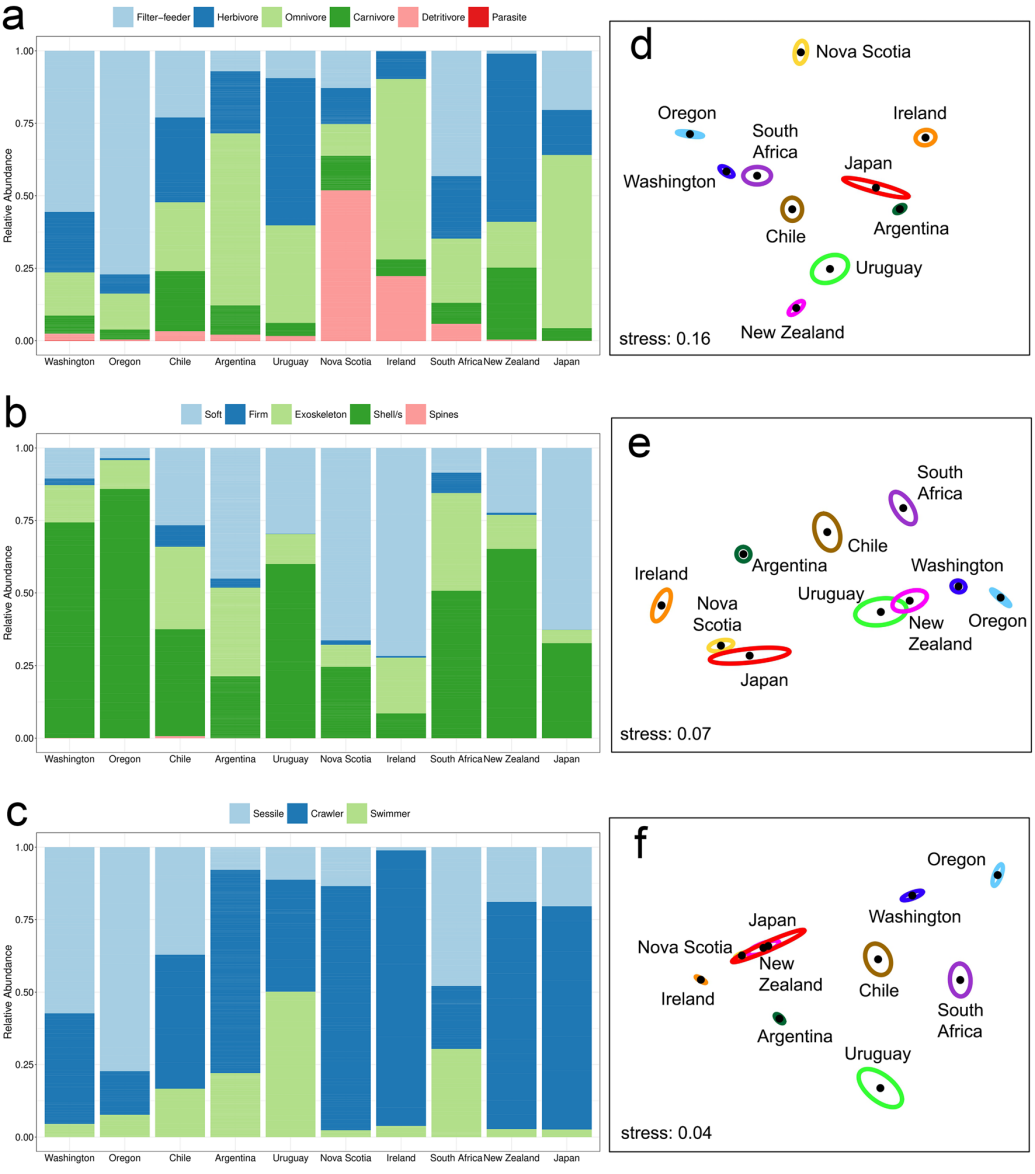
Mean relative abundance of categories of (**a**) trophic level, (**b**) body type, and (**c**) mobility for invertebrate assemblages from rocky-intertidal mussel beds from each surveyed coast and NMDS ordination of centroids (surrounded by standard-error ellipses) for each coast summarizing their functional composition considering categories of (**d**) trophic level, (**e**) body type, and (**f**) mobility.

The functional composition of the associated communities varied among coasts (pseudo *F*_9, 606_ = 47.42, *P* < 0.001), but NMDS revealed overlaps among most coasts (Fig. 8). To identify the trait categories that characterize the associated communities across coasts, we calculated (separately for each trait) the relative density of each trait category for each plot. Almost all of the trait categories were represented on all coasts, the exceptions being detritivores and firm bodies (present on all coasts but Japan), bodies with spines (sea urchins, reported only for Washington and Chile), and parasites (reported only for Washington). The trait categories differed in relative density among the coasts (Fig. 9, Supplementary Table S1), showing some geographic patterns of interest that are described below.

Regarding trophic levels, filter-feeders were relatively most abundant on the eastern Pacific coast (mainly Washington and Oregon and then Chile) and in South Africa, while detritivores predominated on the two coasts surveyed in the northern Atlantic (Nova Scotia and Ireland). Herbivores were relatively most abundant in the southern hemisphere (mainly New Zealand and Uruguay, followed by Chile, Argentina, and South Africa) and Washington. Carnivores were typically less abundant than herbivores, being relatively most abundant in New Zealand, Chile, and Argentina. Omnivores did not show particularly clear geographic patterns. An NMDS ordination of plots based only on the relative density of trophic levels further revealed these regionalities. For instance, the three surveyed eastern Pacific coasts (Washington, Oregon, and Chile) were nearby in the ordination and close to South Africa. In turn, Chile was also close to the other South American surveyed coasts (Argentina and Uruguay). Meanwhile, the two coasts surveyed in the north Atlantic (Nova Scotia and Ireland) coincided in a separate section of the ordination graph (Fig. 9).

Regarding body types, organisms with shells were the most abundant organisms on the eastern Pacific coast (Washington, Oregon, and Chile), South Africa, New Zealand, and Uruguay, while organisms with soft bodies were relatively most abundant in the north Atlantic (Nova Scotia and Ireland), Argentina, and Japan. Organisms with other body types exhibited more variable geographic patterns of abundance. Nonetheless, an NMDS ordination of plots based on the relative density of body types revealed the same basic regionalities described for trophic levels (Fig. 9).

Regarding mobility types, sessile organisms predominated on the eastern Pacific coast (Washington, Oregon, and Chile) and South Africa. Crawlers were best represented in the northern Atlantic (Nova Scotia and Ireland) but were also relatively abundant on distant coasts (New Zealand, Japan, and Argentina). Swimming organisms were best represented in Uruguay. Interestingly, an NMDS ordination of plots based on the relative density of mobility categories showed the same basic regionalities as for trophic levels and body types (Fig. 9).

## Discussion

Using data from temperate coasts from the eastern and western boundaries of the Atlantic and Pacific Oceans from both hemispheres, we studied biogeographic patterns in the taxonomic and functional composition of invertebrate assemblages from rocky-intertidal mussel beds. Remarkably, while the number of taxa identified per coast differed by nearly ten times between the most extreme cases (Washington vs. New Zealand), values were within the same order of magnitude for all coasts excluding Washington (between 26 and 75 taxa). As the datasets available for this study were originally collected for different purposes, the environmental range covered by each survey differed to some extent. This is worthy of consideration because, in rocky intertidal habitats, physiological stress varies across elevations and hydrodynamic physical stress varies horizontally along the shore^32–34^, and species richness often increases with environmental heterogeneity^35–37^. The Washington survey covered the most heterogeneous environmental range, spanning low to high elevations and wave-exposed to more sheltered habitats^38^ (see also Supplementary Note). Thus, this might partially explain why that survey identified more taxa than the other surveys. In any case, the finding that the number of identified taxa was within the same order of magnitude for all of the other coasts reveals a commonality for intertidal mussel beds. Moreover, when taxonomic richness was expressed per unit area (at a spatial resolution of dm^2^), it differed little among coasts even including Washington. This finding indicates that intertidal mussel beds in general are able to sustain comparable levels of taxonomic complexity per unit area. This property is probably related to the common characteristics that dense mussel beds display^12,14,22,39^ (Fig. 1).

The marked difference among coasts in the taxonomic composition of associated communities when viewed at the taxonomic resolution reported by the surveys (often to the species level) was not surprising given the divergent evolutionary paths of biogeographic regions. Nonetheless, some regional patterns were clear, as the plot clusters of Washington and Oregon were nearby in the multivariate ordination, as were those of Chile and Argentina and those of Nova Scotia and Ireland. These regionalities might be primarily determined by oceanography. For instance, the Washington and Oregon coasts are washed by the southward California Current, while the Chilean and Patagonian Argentine coasts are washed by northward currents that branch off the Antarctic Circumpolar Current at the southern tip of South America^40,41^. Similarly, the Nova Scotia and European coasts share a biogeographic history related to trans-Atlantic dispersal of intertidal organisms^42–45^.

Sorting the identified taxa into 28 taxonomic groups revealed basic global commonalities, as amphipods, decapods, gastropods, isopods, and polychaetes were present on all of the surveyed coasts, while anthozoans, barnacles, bivalves, and nemerteans occurred on at least eight coasts. Moreover, using the abundance data for the 28 groups, overlaps in taxonomic composition emerged among most coasts in multivariate space. These findings indicate that the main groups of organisms hosted by rocky-intertidal mussel beds are relatively consistent despite species-specific differences rooted mainly in biogeography. A few regional patterns were also evident, as barnacles had the highest relative abundances on the eastern Pacific coast (Washington, Oregon, and Chile) in addition to South Africa, while oligochaetes and nematodes were relatively most abundant in Nova Scotia and Ireland. Explaining the abundance differences among coasts for the 28 groups is difficult because of the differing environmental range covered by the surveys. In addition, changes in the abundance of even a few species can alter community structure through direct and indirect interspecific interactions that are often unpredictable without experimentation^46^, so snapshot abundance data often cannot reveal the underlying interaction web^47,48^. Interaction webs for communities of primary-space holders (macroalgae and filter-feeders) and their consumers (sea stars, crabs, snails, etc.) are well understood for many rocky shores^49–52^, but this is not the case for the invertebrate assemblages living in intertidal mussel beds. These knowledge gaps represent opportunities for future research.

When we studied functional traits of the associated communities, common patterns among coasts also emerged. The polygons used to calculate functional richness in trait space overlapped greatly among coasts, indicating that similar trait combinations occur in these systems. Moreover, the associated communities had limited differences in standardized functional richness (functional richness per unit area) among most coasts, the exceptions being only Washington and Uruguay, with lower values. Relative to the other coasts, Washington had lower functional richness than taxonomic richness, indicating a higher functional redundancy in Washington. This may result from the high taxonomic resolution of the Washington survey and the high diversity of species in groups low in the taxonomic hierarchy.

While those global commonalities were evident, the relative abundance of trait categories showed regionalities. These patterns were related to those for the taxonomic groups described above, as indicated by particular combinations of trait categories. For instance, the prominence of filter-feeding, shell possession, and sessility on the eastern Pacific coast (Washington, Oregon, and Chile) and South Africa was related to the combined predominance of barnacles and bivalves (excluding the mussels composing the beds) on those coasts. As coastal upwelling is important on those shores^40,41^, the phytoplankton blooms and the detritus of the associated pelagic food webs that are facilitated by upwelling might explain the predominance of such organisms and traits in those mussel beds. Another taxonomic–functional relationship is exemplified by the prominence of detritivory, soft bodies, and crawling in the north Atlantic (Nova Scotia and Ireland), which reflects the predominance of oligochaetes and nematodes on those coasts. On the other hand, the three coasts surveyed in South America (Chile, Argentina, and Uruguay) were always nearby in multivariate space when ordinated using the categories of the three considered functional traits.

In summary, this global study supports the notion that rocky-intertidal mussel beds host invertebrate assemblages that share to a great extent a common set of functional properties. Almost all of the studied trait categories were represented on all surveyed coasts. This is remarkable because the studied coasts differ greatly in taxonomic composition at the taxonomic resolution (often species) used by the surveys. Grouping the identified taxa at higher taxonomic levels, however, also revealed mussel beds as hosts of a common set of organisms globally, as amphipods, anthozoans, barnacles, bivalves, decapods, gastropods, isopods, nemerteans, and polychaetes were present on 80–100% of the surveyed coasts. Where mussel beds from different shores differ the most is in the relative abundance of taxonomic groups and the corresponding trait categories.

From a parallel perspective, this study also provides evidence that a given type of foundation species can host functionally similar communities at the level noted above even when the underlying species pool differs for biogeographic reasons. This conclusion should help researchers in two ways. First, unstudied foundation species could be expected to host communities similar in function to those hosted by the same kind of foundation species that has been studied elsewhere. Second, if foundation species continue to be lost because of anthropogenic factors^5–8^, this knowledge will aid restoration efforts that attempt to recreate specific functions lost in an ecosystem.

Global studies of functional diversity are being conducted for an increasing number of organisms, such as mammals^53^, spiders^54^, freshwater fish^55^, corals^56^, and reef fish^57,58^, for example. While a diversity of geographic patterns is being encountered, our study’s conclusions are broadly similar to some of those studies. For example, McLean et al.^57^ found that trait composition in reef fish assemblages is globally unrelated to taxonomic diversity and instead is mainly driven by environmental filtering that works similarly across oceans. For rocky-intertidal mussel beds, their main structural and functional properties are seemingly the drivers of the common broad taxonomic and functional patterns in their associated communities. Globally common functional patterns are not universal for all groups of organisms, however, as the functional richness of coral assemblages differs greatly across biogeographic regions^56^. In an era of increasing ecological change, these dissimilar results highlight the need to better understand the main drivers of functional properties of communities. Future refinements of studies done for mussel beds could include conducting contemporary surveys on various shores (which will require several teams) with the same sampling effort to exclude potential sources of variation such as seasonality, interannual variability, recent biological invasions, and differences in sampling methodology. Meanwhile, the present study will hopefully stimulate a number of experimental studies to unravel the drivers of taxonomic and functional properties of communities associated to rocky-intertidal mussel beds.

## Methods

### Species abundance datasets

To make this study as global as possible, we used datasets describing the abundance of invertebrate taxa found in rocky-intertidal mussel beds from the NW, SW, NE, and SE coasts of the Pacific and Atlantic Oceans, as those are temperate coasts where intertidal mussels thrive (Fig. 2). Because few of such datasets were publicly available when we began this study (likely because data publishing is a recent practice), we supplemented the few published datasets with datasets provided by contributors who originally produced them for other purposes. We identified these sources using the Web of Science and Google Scholar and we used all of the datasets that became available to us after maximizing attempts to contact these sources. Table 1 lists the used data sources, while the datasets themselves are provided in Supplementary Table S1. The main properties of the coasts where these datasets came from, the collection dates, the names of the mussel species that structured the beds, and a validity assessment for these sampling units are provided in the Supplementary Note. All scientific names have been updated according to the World Register of Marine Species^59^. Essentially, these surveys measured the abundance of species of the associated communities found in replicate plots collected in dense stands of living mussels from rocky intertidal habitats. Table 1 also specifies the number and size of plots used by each survey. Among the collected mussel stands, those surveys often found seaweeds, barnacles, and bare rock, as is typical of temperate rocky seashores. The studies that produced those datasets are listed in the Supplementary Note and provide additional methodological details in the corresponding methods sections.

### Global taxonomic comparison

We taxonomically compared the associated communities among coasts using univariate and multivariate approaches. For each plot, we calculated the number of invertebrate taxa identified by each study (taxonomic richness). Because plot size differed across surveys (Table 1) and taxonomic richness increased linearly with plot size (*r* = 0.88, *P* < 0.001, *N* = 616 plots), for comparisons we standardized taxonomic richness by dividing the number of identified taxa in a plot by the plot’s area. We compared standardized taxonomic richness among coasts through a permutational one-way analysis of variance (ANOVA) using 4999 random permutations.

We then compared the taxonomic composition of coasts by considering both the identity and abundance of the identified taxa. Beforehand, we calculated for each plot the density of each identified taxon by dividing its abundance (number of organisms) in the plot by the plot's area. Using the density values, we compared taxonomic composition among coasts through a permutational multivariate analysis of variance (perMANOVA) using 4999 random permutations. As significant differences were encountered (see “Results”), we ascertained how coasts related to one another through a nonmetric multidimensional scaling (NMDS) ordination based on Bray–Curtis dissimilarities between plots^60^.

Since those analyses used density data for taxa identified at the lowest possible taxonomic level by each survey, the datasets were dominated by density values for species (Table 1, Supplementary Table S1). This property of the data likely explains the clear differences in taxonomic composition among coasts (see “Results”). Therefore, we evaluated if taxonomic composition would be more similar among coasts if analyzed at a coarser taxonomic resolution, under the notion that species within broad groups are often ecologically similar, which would allow such groups to be represented more similarly across mussel beds given the common properties of mussel beds. We grouped the identified taxa into 28 taxonomic groups (Supplementary Table S1). Then, using density values calculated for those groups, we compared group taxonomic composition among coasts through a perMANOVA using 4999 random permutations and an NMDS ordination based on Bray–Curtis dissimilarities between plots.

### Global functional comparison

To infer functional properties of the associated communities, we used three functional traits: trophic level, body type, and mobility. We considered six possible categories for trophic level (filter-feeder, herbivore, carnivore, omnivore, detritivore, and parasite), five categories for body type (soft body without external protections, firm body without external protections, body with an exoskeleton, body with calcified shell/s, and body with spines), and three categories for mobility (sessile, crawler, and swimmer). These traits were chosen to cover important functional aspects such as the ability to process different kinds of food, the ability to resist physical stress, and movement abilities. We assigned each identified taxon to the corresponding category of each trait using information from online databases and the scientific literature^59,61,62^. The trait categories assigned to each identified taxon are specified in Supplementary Table S1.

Once the identified taxa were assigned to the corresponding trait categories, for each plot we calculated functional richness based on Gower distances between taxa using their values (1 or 0) for each trait category^63^. Because functional richness closely increased linearly with taxonomic richness (*r* = 0.93, *P* < 0.001, *N* = 616 plots) and taxonomic richness increased with plot size, which varied among surveys (see above), for comparisons we standardized functional richness by dividing the value obtained for each plot by the plot’s area. We compared standardized functional richness among coasts through a permutational one-way ANOVA using 4999 random permutations.

Finally, we compared coasts based on the functional composition of the associated communities, which refers to a combined measure of the identity and abundance of each trait category. First, we calculated for each plot, and separately for each of the three functional traits, the relative abundance of each trait category as the sum of the values of relative abundance of the identified taxa that were assigned to each trait category. Then, using those values, we compared functional composition among coasts through a perMANOVA using 4999 random permutations. As significant differences were found (see “Results”), we evaluated how coasts related to one another through an NMDS ordination based on Bray–Curtis dissimilarities between plots.

We conducted the data analyses using the FD^63^, RVAideMemoire^64^, and vegan^65^ packages in R version 4.0.2.

### Supplementary Information

Supplementary Information.

Supplementary Table S1.

## Data Availability

The entire dataset used for this study is provided in Supplementary Table S1.
