# Supplementary material for: Global taxonomic and functional patterns in invertebrate assemblages from rocky-intertidal mussel beds
Source: Sci Rep. 2024 Jan 2;14:26. doi: 10.1038/s41598-023-50549-8 (PMC10761853; doi:10.1038/s41598-023-50549-8)
Supplement: Supplementary file 1 — Supplementary Information. [file 41598_2023_50549_MOESM1_ESM.pdf]

Global taxonomic and functional patterns in invertebrate assemblages from rocky-intertidal mussel beds

Nicole M. Cameron, Ricardo A. Scrosati, Nelson Valdivia, and Zechariah D. Meunier

Supplementary Note

Main characteristics of the locations where the species abundance datasets used for this study came from.

NE Pacific

Washington, USA [1]

The Washington dataset was published in a Ph.D. dissertation [1]. The data were collected at a wave-exposed site in Tatoosh Island (48.4, -124.733333) and at a moderately exposed site at Shi-Shi Beach (48.283333, -124.683333). At these sites, mussel beds are composed of *Mytilus californianus*. In July 1974 and July 1976, the author collected 39 plots at Tatoosh Island and 15 plots at Shi-Shi Beach (1000 cm^2) with the associated invertebrates and underlying sediment. Samples were collected at the high, middle, and low intertidal zones. Preservation was done via freezing, fixing in 10 % formalin (buffered with methenamine), and stored in 80 % ethanol (with 5 % glycerine). Species identifications were done for algae, invertebrates, and fish over 1 mm in size.

Oregon, USA (Zechariah D. Meunier, unpublished data provided for this article)

In Oregon, the samples were collected at four sites: Fogarty Creek (44.837, -124.0589), Boiler Bay (44.8317, -124.0608), Strawberry Hill (44.2499, -124.1153), and Port Orford Heads (42.7441, -124.5143). At these sites, mussel beds are composed primarily of *Mytilus californianus* and occasionally *Mytilus trossulus*. Between May and June 2020, the author collected 10 plots at Fogarty Creek and Port Orford Heads and 5 plots at Boiler Bay and Strawberry Hill (100 cm^2). Samples were collected between the middle and low intertidal zones at varying wave exposures. Invertebrates over 0.5 mm in size were preserved in 70 % ethanol and identified to the lowest possible taxonomic level with a dissecting microscope using identification guides [2–4].

SE Pacific

Chile (Nelson Valdivia, unpublished data provided for this article)

These data were collected to do the study by Valdivia and colleagues [5]. At a moderately exposed site near Coquimbo (30.05, -71.466667), experimental *Perumytilus purpuratus* patches (225 cm^2) were established and collected between December 2002 and June 2003. The authors were interested in the colonization of mussel beds by invertebrates, so plots were deployed for different periods of time. For our global study, we only used the abundance data for plots deployed for 4 months or longer because of the authors' assertion that after 3-4 months the associated invertebrate community was representative of natural beds. Mussels and their associated organisms were collected and transported in plastic bags or jars to the lab. Samples were fixed with 7 % formalin. Collections were washed over a 0.5 mm sieve and all organisms were identified to the lowest possible taxonomic level with a dissecting and binocular microscope.

SW Atlantic

Argentina (Lorena P. Arribas, unpublished data provided for this article)

A large part of the data available for Argentina for our study was used in a study published by Arribas and colleagues [6]. Mussel beds composed of *Brachidontes rodriguezii* and *Perumytilus purpuratus* were collected between autumn 2009 and winter 2011 at six sites spanning the provinces of Buenos Aires and Río Negro: Santa Elena (-37.51, -57.30), Mar del Plata (-38.02, -57.53), El Espigón (-41.12, -63.00), La Lobería (-41.16, -63.03), Playa Los Suecos (-41.70, -65.02), and Punta Colorada (-41.12, -65.02). Two hundred and eighty-eight plots were collected with a core measuring 10 cm in diameter (78.5 cm^2) and organisms were fixed with 96 % ethanol. Any invertebrate over 0.5 mm was identified to the lowest possible taxonomic level with a stereoscopic microscope.

Uruguay (Ana I. Borthagaray, unpublished data provided for this article)

The data from Uruguay were collected to do a study published by Borthagaray and Carranza [7]. Three sites of varying wave exposure were surveyed near Cerro Verde (-33.950, -53.500). Twenty-nine patches (400 cm^2) dominated by *Brachidontes rodriguezii* but that also included *Mytilus edulis platensis* and *Perna perna* were collected. The authors also collected samples of non-mussel patches and subtidal communities that were not considered for our study. The organisms from mussel patches were fixed and identified in the laboratory.

NW Atlantic

Nova Scotia, Canada [8]

This dataset was published in a data paper [8] and was originally collected to do a study published by Arribas and colleagues [9]. Six sites along the Atlantic coast of Nova Scotia were surveyed, including three wave-exposed sites named Coote Rock area (45.182894, -61.353258), Crystal Crescent Beach (44.447372, 63.622214), and Kejimkujik (43.818614, -64.834747) and three wave-sheltered sites named Webber Cove (45.188347, -61.354853), Casino Nova Scotia (44.651814, -63.573731), and Halifax Harbourfront (44.648181, -63.570303). At these sites, mussel beds are composed of *Mytilus edulis* and *Mytilus trossulus* [10,11]. Between early September and early October 2012, the authors collected 15 plots (100 cm^2) fully covered by mussels at each site including the associated invertebrates. Preservation was done via freezing and identification was conducted with a microscope and field guides for organisms over 0.5 mm in size.

NE Atlantic

Ireland (Nessa E. O'Connor, unpublished data provided for this article)

These data were collected to do a study published by O'Connor and Crowe [12]. Experimental patches of *Mytilus edulis* were created in March 2003 on the east coast of Ireland: Clogherhead in Louth County (53.792, -6.218) and Rush in Dublin County (53.523, -6.082). The authors tested for differences in the associated communities based on the size of mussels in the bed. Although these beds were experimental, the authors stated that they were representative of natural beds. Six months after the experiment began, 7 patches from Louth County and 8 from Dublin County were collected, washed through a 0.5-mm sieve, and the associated invertebrates were identified to the lowest possible taxonomic level according to the European Register of Marine Species.

SE Atlantic

South Africa (Alan N. Hodgson, unpublished data provided for this article)

This dataset was collected to do a study published by Hodgson and colleagues [13]. In the Knysna Estuary, 100-cm^2 plots dominated by *Mytilus galloprovincialis* were collected at six sites: The Heads (-34.080442, 23.062427), Featherbed (-34.075589, 23.055207), Leisure Isle (34.070825, 23.061558), Thesen Wharf (-34.048142, 23.046967), Marina (-34.048142, 23.046967), and Railway Bridge (-34.042524, 23.032462). We determined the coordinates of these sites from a combination of figure 1 in [13] and Google Maps. Despite being described as an estuary, the tides, temperature, and salinity are similar to the adjacent ocean. Ten plots were collected at each site between summer (15 February 2018) and autumn (15 April 2018) from the mid- to low intertidal zone. Samples were transported in plastic bags to the laboratory where they were identified to the lowest possible taxonomic level.

SW Pacific

New Zealand (Andrew R. Davis, unpublished data provided for this article)

This dataset was collected to do a study published by Davis and colleagues [14]. Ten patches of *Perna canaliculus* (100 cm^2) were collected in January 2014 at each of two sites in northern New Zealand: Tauroa Peninsula (-35.168889, 173.108889) and Shipwreck Bay (-35.176944, 173.1325) at an intertidal elevation of ca. 0.2 m. At each site, five patches were collected from shallow tidepools and five from emergent rock. Based on the marked overlap in species composition between both habitat types according to the authors, we included all plots for our study. Samples were frozen and transported to the laboratory where invertebrates over 0.5 mm in size were identified to the lowest possible taxonomic level.

NW Pacific

Japan [15]

This dataset was published by Tsuchiya and Nishihira [15]. Patches of a species identified as *Mytilus edulis* were collected by the Marine Biological Station of Tohoku University (40.916667, 140.833333). Samples were collected in June of an unspecified year before 1985 and the associated invertebrates were identified to the lowest possible taxonomic level. Twenty-four plots of varying size (0.4 cm^2 to 521.3 cm^2) were collected for the original study, but we only used data for the largest plots to ensure similar plot sizes relative to the other surveys considered in our study. When selecting what plot sizes to consider from the Japan survey, a careful balance was needed to keep a large enough sample size without unduly inflation of density values due to small patch sizes.

References

[1] Suchanek, T. H. The *Mytilus californianus* community: Studies on the composition, structure, organization and dynamics of a mussel bed. Ph.D. dissertation (University of Washington, 1979).

[2] Kozloff, E. N. *Marine invertebrates of the Pacific Northwest.* University of Washington Press (1987).

[3] Lamb, A. & Hanby, B. P. Marine life of the Pacific Northwest: A photographic encyclopedia of invertebrates, seaweeds, and selected fishes. Harbour Publishing (2005).

[4] Carlton, J. T. *The Light and Smith manual: Intertidal invertebrates from central California to Oregon.* University of California Press (2007).

[5] Valdivia, N., Buschbaum, C. & Thiel, M. Succession in intertidal mussel bed assemblages on different shores: Species mobility matters. *Mar. Ecol. Prog. Ser.* **497**, 131–42 (2014).

[6] Arribas, L. P., Bagur, M., Klein, E., Penchaszadeh, P. & Palomo, M. G. Geographic distribution of mussel species and associated assemblages along the northern Argentinean coast. *Aq. Biol.* **18**, 91–103 (2013).

[7] Borthagaray, A. I. & Carranza, A. Mussels as ecosystem engineers: their contribution to species richness in a rocky littoral community. *Acta Oecol.* **31**, 243–250 (2007).

[8] Scrosati, R. A., Arribas, L. P. & Donnarumma, L. Abundance data for invertebrate assemblages from intertidal mussel beds along the Atlantic Canadian coast. *Ecology* **101**, e03137 (2020).

[9] Arribas, L. P., Donnarumma, L., Palomo, M. G. & Scrosati, R. A. Intertidal mussels as ecosystem engineers: Their associated invertebrate biodiversity under contrasting wave exposures. *Mar. Biodiv.* **44**, 203–211 (2014).

[10] Tam, J. C. & Scrosati, R. A. Mussel and dogwhelk distribution along the NW Atlantic coast: Testing predictions derived from the abundant-centre model. *J. Biogeogr.* **38**, 1536–1545 (2011).

[11] Tam, J. C. & Scrosati, R. A. Distribution of cryptic mussel species (*Mytilus edulis* and *M. trossulus*) along wave exposure gradients on northwest Atlantic rocky shores. *Mar. Biol. Res.* **10**, 51–60 (2014).

[12] O'Connor, N. E. & Crowe, T. P. Biodiversity among mussels: Separating the influence of sizes of mussels from the ages of patches. *J. Mar. Biol. Assoc. U. K.* **87**, 551–557 (2007).

[13] Hodgson, A. N., Smith, F., Smith, P. & Claassens, L. Macrofauna associated with intertidal mussel beds in the Knysna estuarine embayment, South Africa. *African Zool.* **56**, 44–57 (2021).

[14] Davis, A. R., Walls, K. & Jeffs, A. Biotic consequences of a shift in invertebrate ecosystem engineers: Invasion of New Zealand rocky shores by a zone-forming ascidian. *Mar. Ecol.* **39**, e12502 (2018).

[15] Tsuchiya, M. & Nishihira, M. Islands of *Mytilus edulis* as a habitat for small intertidal animals: Effect of island size on community structure. *Mar. Ecol. Prog. Ser.* **25**, 71–81 (1985).
